# Thermoregulation in wheelchair tennis—How to manage heat stress?

**DOI:** 10.3389/fphys.2015.00175

**Published:** 2015-06-02

**Authors:** Olivier Girard

**Affiliations:** Department of Physiology, Faculty of Biology and Medicine, Institute of Sport Sciences, University of LausanneLausanne, Switzerland

**Keywords:** wheelchair tennis, wet bulb globe temperature, thermoregulation, spinal cord injury (SCI), heat stress

Founded in 1976 and having become a full medal sport at the 1992 Barcelona Paralympics, the popularity of wheelchair tennis continues to grow. With the exception of the “double-bounce rule,” wheelchair and able-bodied tennis follow the same rules. Most of tennis matches are played in cool outdoor conditions or climate-controlled indoor venues. Nonetheless, it is common for top-level players to be exposed to hot (>30°C) and/or humid (>70% rH) conditions during competition or training [Wet Bulb Globe Temperature (WBGT) of 28°C or greater]. At the 2009 Australian Open championship, Australia's former world No 1 Daniela Di Toro, spoke about the high court temperatures (often above 40°C)—“*You've got the direct heat overhead as well as radiant heat all around you that has been absorbed by the court and your chair, and it really is extremely full-on.*” Heat stress may not only threaten the quality of play, but could potentially pose a risk to the players' health. Detailed description of thermal, physiological and perceptual responses of able-bodied players to simulated competitions can be found in the literature (Fernandez Fernandez et al., 2006), while the investigations that have involved Paralympic tennis players are rare. Evaluating the specific game requirements is a prerequisite of more thoroughly understanding the physiology of wheelchair tennis. This task is, however, not easy due to numerous modulating internal (i.e., variety of physiological impairment, competitive standard, playing style, gender and body composition) and external (i.e., environmental conditions, ball type and court surface) factors of tennis match intensity, in addition to high individual variability in physiological responses to match play. Moreover, it is difficult to develop and implement universal safety standards and guidelines to account for all of the environmental scenarios. Well aware of potential health risks, the International Tennis Federation (ITF) medical commission has implemented policies for effectively reducing heat illness risk to safeguard wheelchair tennis players' health when competing in environmentally challenging conditions (http://www.itftennis.com/media/166656/166656.pdf). In addition to these existing procedures in wheelchair tennis the question still exist as to what preventive countermeasures can be implemented when it gets hot.

## Current regulations/guidelines for preventing heat injury in wheelchair tennis

To date, different rules and regulations are dictated by various tennis organizations (ATP, WTA, ITF, and Grand Slams) and even within the same governing body (ITF) recommendations for preventing heat injury are not uniformly applied between men, women, juniors or wheelchair tennis players. Curiously, no heat rule prevails in general in the able-bodied ITF men's circuit or ITF seniors. In extraordinary circumstances only, the referee in consultation with the tournament director and/or ITF supervisor may suspend play or adjust a tournament's schedule in the case of darkness, weather or adverse court conditions. In other categories including ITF Juniors, ITF Women's circuit and Fed Cup, the starting time of matches scheduled for play may be delayed where, in the opinions of tennis officials, extreme weather conditions are likely to come into effect. For WTA matches already in progress where WBGT ≥ 30°C, a 10 min break in play between the second and third sets has recently been implemented, with immediate suspension of play in extreme conditions (WBGT ≥ 32°C). The current rules for wheelchair tennis (ITF) are:

- 28°C ≤ WBGT ≤ 30°C—15-min break between the second and third sets.
- 30°C ≤ WBGT ≤ 32°C—Suspension of play at the end of the set in progress and will not resume until WBGT falls below 30.1°C.
- WBGT ≥ 32°C—Immediate suspension of play that will not resume until WBGT falls below 30.1°C.

WBGT is an index calculated from wet-bulb, dry-bulb, and black-globe temperatures that is commonly used to quantify heat stress in occupational, military and sports context. Despite not being an actual representation of players' heat strain, WBGT is largely recommended by the most prestigious sport organizations (IOC) and endorsed by leading international sports federations (FIFA, IAAF, ITF) (Bergeron et al., 2012; Mountjoy et al., 2012). These recommendations that are based on WBGT cut-offs address the potential risks for a broad range of players without taking into consideration their individual characteristics (e.g., fitness level, acclimatization). Moreover, with obvious variation in worldwide climatology, the use of this index that neglects the influence of cloud cover (affecting the intensity of solar radiation) and wind speed is not without limitations (Brocherie et al., 2014). Given that metabolic rate determines exercise-induced heat strain in sports, regardless of the environmental conditions (Brotherhood, 2008) it is evident that heat dissipation cannot be predicted from WBGT measurements. This implies that, in decision on when to suspend the play, quantifying metabolic heat gain might be a more suitable approach rather than using universal fixed cut-offs based solely on the WBGT index or air temperature.

## What does research tell us about able-bodied tennis in the heat?

Competitive match-play tennis in the heat leads to significantly greater levels of thermal, physiological and perceptual strain compared to cooler conditions where steady core temperatures, (i.e., fluctuating around 38.5°C), are recorded (Périard et al., 2014). In hot ambient conditions core temperatures above 39°C have been reported during play (Bergeron et al., 2007; Morante and Brotherhood, 2008; Périard et al., 2014), suggesting that contrary to temperate environments, the efficiency of autonomic (e.g., cutaneous vasodilatation, active sweat secretion) and behavioral (e.g., adjustments in play and recovery) thermoregulatory mechanisms cannot successfully regulate the rate of heat gain. For example, when competing in hot conditions (~37°C, ~36% relative humidity, ~34°C WBGT and ~0.5 m/s wind velocity), players' rectal and thigh skin temperatures increased to ~39.4°C and ~37.5°C, respectively, leading to exacerbation of the perception of effort and thermal perception (Périard et al., 2014).

Body heat storage capacity, influencing changes in core temperature, is determined by the cumulative imbalance between metabolic heat gain and net heat loss to the environment (Cramer and Jay, 2014). Heat balance is easily disturbed as a result of changes in metabolic heat gain due to intense physical activity and/or exposure to a warmer environment. Thermoregulatory differences between men, women and children wheelchair tennis players may relate to substantial inter-individual variability (Goosey-Tolfrey et al., 2008b) and/or the influence of independent factors (e.g., body mass or body composition) on thermoregulatory outcomes during exercise (Havenith et al., 1998). Generally speaking, players with larger body and muscle masses are characterized by greater heat gain that must be dissipated to maintain “safe” body temperatures. An increased thermal strain in hot playing conditions, that is also associated with warmer skin temperature readings (Morante and Brotherhood, 2008), induces behavioral adaptations, as thermal sensation become less comfortable. For instance, in able-bodied tennis, adjustment of match-play characteristics in hot conditions comes from evidence of shorter points durations (Morante and Brotherhood, 2008) or longer rest periods between points (Périard et al., 2014), in turn reducing the effective playing time (i.e., the percentage of total match time spent with the ball in play).

## Thermoregulation in wheelchair tennis

Few studies have examined thermoregulatory responses during the wheelchair game (Veltmeijer et al., 2014; Griggs et al., 2015), yet the prevailing view is that individuals with a spinal cord injury (SCI) demonstrate impaired thermoregulatory function compared to able-bodied peers. This puts them at a higher risk (proportional to the injury level) of developing heat illnesses (Price, 2006). In one such study, thermoregulatory responses in wheelchair tennis players with and without a SCI were compared during a 45 min tennis match (WBGT = 18–20°C) (Veltmeijer et al., 2014). Confirming previous laboratory findings (Price, 2006), increases in core temperature were larger in the SCI (+0.6 ± 0.1°C; *n* = 2) compared to the non-SCI (+0.3 ± 0.1°C; *n* = 4) players, whereas mean skin temperature (30–31°C), match characteristics and exercise intensity were similar between groups. The common view is that regulation of body temperatures in SCI players is impaired because of the loss of normal blood-flow regulation via the central nervous system and by the inability to sweat or shiver below the neurological level (Webborn, 1996). In addition to disrupted autonomic nervous system function, loss of skin temperature sensation (i.e., smaller body surface area available for sweating and therefore restrictive evaporative cooling of the skin) also characterizing SCI players shouldn't be overlooked. Such impaired ability to detect thermal injury would indicate that individuals with spinal cord-related lesions (T6 and above) are more prone to heat illness due to increased lower body heat storage (Veltmeijer et al., 2014).

## What preventive countermeasures are potentially useful to minimize heat strain during the wheelchair game?

Cooling strategies (Griggs et al., 2015), structured heat acclimatization (Castle et al., 2013) and hydration interventions (Goosey-Tolfrey et al., 2008a) are well-established methods for improving exercise performance in SCI players competing in warm-to-hot conditions. These strategies are helpful in reducing thermal and cardiovascular strain, while ameliorating perceptual responses and improving exercise tolerance. In one case study, for instance, one female player, a member of the Great Britain national team involved in the preparation of Athens 2004 Paralympics, underwent a simulated 1-h wheelchair tennis protocol in hot conditions (30°C, 54% rH) (Diaper and Goosey-Tolfrey, 2009). Peak sprinting speeds were better maintained when 30 min precooling (cooling vest) was combined with head and neck cooling (cooling hats and neck bands) during exercise. This occurred together with an improvement in thermal sensation and slower rise of aural temperature toward the latter exercise stages. Partial heat acclimation may also be beneficial for athletes with an SCI, as demonstrated by lower resting core temperature and increased plasma volume (yet without any improvement in sweat response) following 7 days of heat acclimation (Castle et al., 2013).

Creating universal guidelines on preventive countermeasures for wheelchair tennis players is challenging, as success will both depend on the environmental conditions of the competition venue and the level of SCI. Hence, individuals with high-level lesions (tetraplegia) possess a greater thermoregulatory impairment (i.e., impaired ability to dissipate heat due to a larger body surface area that cannot actively regulate body temperatures) than individuals with lower-level lesions (paraplegia). Reportedly, tetraplegic compared to paraplegic athletes display disproportionate increases in core temperature and body heat storage in spite of producing similar external work during the completion of an intermittent-sprint protocol in cool conditions (Griggs et al., 2015). Variation in the degree of sweating and blood flow redistribution between SCI individuals implies that preventive strategies should be individualized. For instance, it is recommended to determine the acclimatization response of each SCI player individually by recording the changes in haematocrit concentration after a heat-response test (Racinais et al., 2012). Furthermore, implementation of more “aggressive” cooling strategies (i.e., combined methods and/or larger surface area coverage) likely improves thermal comfort and eventually functional capacity (Minett et al., 2011).

Although non-SCI players may be able to cool down during the rest periods in between rallies, games or sets, the SCI players might not be able to do so as effectively. Well aware that SCI tennis players have an elevated risk of developing heat illness, tournament organizers and governing bodies have the responsibility to support the implementation of countermeasures to prevent hyperthermia (e.g., allowing extra time for cooling the body). Investigating the thermal effect of a 10-min break between the 2nd and 3rd sets during a game raising core temperature to ~1.3°C after ~80 min of play showed encouraging results; i.e., core temperature decreased by 0.25 ± 0.20°C for 6 of the 7 able-bodied women participants including all whose temperatures were above 39.0°C (Tippet et al., 2011). Whether there is enough scientific merit of using these results to support the implementation of 15 min break between the second and third set when the WBGT hits 28°C in the wheelchair game is still unknown. Perhaps ingestion of an intestinal pill or a radio-pill suppository for continuous monitoring of core temperature (e.g., gastrointestinal and rectal measurements, respectively) would enable informed decision on when to suspend play for games scheduled in hot ambient conditions. Simultaneous computations of deep-body temperature at various measurement sites (e.g., sublingual, aural, esophagus) could also be obtained by trained users (Taylor et al., 2014). While research has shown that it is possible to achieve greater cooling ability in individuals with SCI through the use of specific cooling garments (ice-packed vests; Webborn et al., 2005), the implementation of such strategies (i.e., at least during play) remains a challenge within the current laws that govern wheelchair tennis.

## Conclusion

Substantial inter-individual variability that modulate thermal strain certainly represent barriers to develop uniform guidelines for minimizing heat stress during wheelchair tennis competitions. A more appropriate approach, than using fixed WBGT cut-off values, would probably mean that the play must be stopped when the body cannot compensate for the environmental conditions (i.e., cardiovascular drift). As of today, however, the internal metabolic load cannot be easily quantified, but deep core temperature can, to enable individual recommendations on when it is too hot to train or compete safely. Therefore, SCI players are strongly encouraged to implement a range of preventive strategies (e.g., acclimation, cooling, hydration), for instance in view of the 2016 Paralympics in Brazil, to more efficiently regulate their body temperatures. Supporting behavioral thermoregulatory strategies in wheelchair tennis would also imply adjustments in play and/or recovery. In doing so, however, it is crucial that the “game momentum” is not disrupted so as to preserve its popularity.

### Conflict of interest statement

The author declares that the research was conducted in the absence of any commercial or financial relationships that could be construed as a potential conflict of interest.
